# Elucidation of the Genome of Bradyrhizobium sp. Strain USDA 3456, a Historic Agricultural Diazotroph from Cowpea (Vigna unguiculata)

**DOI:** 10.1128/MRA.00812-19

**Published:** 2019-08-15

**Authors:** Richard Allen White, Jeffrey S. Norman, Emily E. Mclachlan, Joseph P. Dunham, Aaron Garoutte, Maren L. Friesen

**Affiliations:** a Department of Plant Pathology, Washington State University, Pullman, Washington, USA; b RAW Molecular Systems (RMS), LLC, Spokane, Washington, USA; c Australian Centre for Astrobiology, University of New South Wales, Sydney, NSW, Australia; d Department of Plant Biology, Michigan State University, East Lansing, Michigan, USA; e SeqOnce Biosciences, Inc., Pasadena, California, USA; f Department of Crop and Soil Sciences, Washington State University, Pullman, Washington, USA; University of Southern California

## Abstract

Bradyrhizobium sp. strain USDA 3456 is a historic strain from the United States Department of Agriculture (USDA) Agricultural Research Service (ARS) National *Rhizobium* Germplasm Collection isolated from Vigna unguiculata (cowpea) in 1966. Strain USDA 3456 has been utilized in global agricultural applications, including improving soil nitrogen fertility. The draft genome sequence here provides a genetic reference of a novel diazotroph.

## ANNOUNCEMENT

The rhizosphere microbiome is one of the most dynamic interfaces on Earth (1–3), with diazotrophs fixing biologically required nitrogen (4, 5). Some diazotrophs can perform symbiotic nitrogen fixation (SNF) by forming root nodules with compatible plant hosts typically in the family Fabaceae, where SNF is selected for by the plant, resulting in fitness alignment (4, 5), or they can fix nitrogen while free-living in soil, aquatic, or other habitats (6).

Bradyrhizobium sp. strain USDA 3456 was isolated from a Vigna unguiculata (cowpea) nodule from Wisconsin in 1966 (7). Field trials with USDA 3456 in peanut (Arachis hypogaea) suggest that it possesses high phosphorus solubilization and moderate indole acetic acid (IAA) production (7, 8). Inoculation of USDA 3456 in peanut crops over two 75-day field seasons resulted in nitrogenase activity of 18 to 19 μmol C_2_H_4_/g^−1 ^(nodule dry weight)/h (8). USDA 3456 can be coinoculated with other plant growth-promoting bacteria, resulting in a synergistic effect on crop production (8).

A lyophilized culture of *Bradyrhizobium* strain USDA 3456 was obtained from the National Rhizobium Germplasm Collection (NRGC) (https://data.nal.usda.gov/dataset/usda-ars-national-rhizobium-germplasm-collection). A single colony was inoculated in AG broth culture (25 ml) at 30°C at 200 rpm to obtain biomass for DNA extraction (9).

DNA was extracted using the MasterPure DNA extraction kit (Epicentre, Madison, WI, USA) following the manufacturer’s protocols. A SeqOnce RhinoSeq kit was used to prepare libraries (https://seqonce.com/rhinoseq/). Libraries were quantified and sequenced on a HiSeq 4000 instrument in a 150-bp paired-end read format at the Michigan State University Research Technology Support Facility (RTSF).

Default parameters were used for all software unless otherwise specified, but versions of software are provided. Illumina sequencing data were quality filtered and decontaminated using ATLAS (version 1.0) (10). The resulting cleaned reads from ATLAS (1,815,290 paired-end reads) were then assembled with Unicycler (version 0.4.7) using default Illumina assembly parameters (11).

The Unicycler final genome assembly for strain USDA 3456 is 107 contigs, with a genome size of 9,771,557 bp, a G+C content of 63.57%, and an *N*_50_ value of 324,457 bp. We estimated completeness and contamination of the draft genome using CheckM (version 1.0.12); the genome was 100% complete with 0% contamination (12). Eight contigs that were <200 bp were removed for submission to GenBank, including the version discussed here. All assemblies are publicly available at Open Science Framework (OSF) for the genome. Prokka (version 1.13.3) with the -rfam flag annotated the genome to obtain rRNAs and transfer-messenger RNAs (tmRNAs) (13). The annotation from Prokka predicted 49 tRNAs, 1 tmRNA, 30 noncoding RNAs (misc_RNAs), 1 copy of 5S-16S-23S operons, 0 CRISPRs, and 9,142 protein-coding genes.

*Bradyrhizobium* sp. strain USDA 3456 is a versatile diazotroph that has been used in agricultural applications for greater than half a century (7). We provide this high-quality draft genome sequence as a template for further metabolic engineering and synthetic biology applications for sustainable agriculture (14, 15).

### Data availability.

This whole-genome shotgun project has been deposited at DDBJ/ENA/GenBank under the accession number VIDU00000000. The version described in this paper is the first version, VIDU01000000. Raw data, contigs, and annotations for this genome can be found at OSF (https://osf.io/5tx6c/), and code used to generate the assembly can be found at www.github.com/friesenlab/Bradyrhizobium_USDA3456.
